# Retraction: Photoactivatable Hsp47: A Tool to Regulate Collagen Secretion and Assembly

**DOI:** 10.1002/advs.202304457

**Published:** 2023-08-15

**Authors:** Essak S. Khan, Shrikrishnan Sankaran, Julieta I. Paez, Christina Muth, Mitchell K. L. Han, Aránzazu del Campo

## Abstract

*Adv. Sci*. **2019**, *6*, 1801982

DOI: 10.1002/advs.201801982

The above article, published online on May 3, 2019, in Wiley Online Library (https://doi.org/10.1002/advs.201801982), has been retracted by agreement between the authors, the journal Editor‐in‐Chief Kirsten Severing, and Wiley‐VCH GmbH. The retraction has been agreed on following concerns raised by a third party and a subsequent investigation by the corresponding authors. Data depicted in Figure 4 and Figure 5 could not be reproduced in follow‐up experiments. Therefore, the conclusions associated with those figures in the article are considered invalid.

E.S.K. participated in the study design, performed measurements, analyzed the data, compiled the figures and participated in manuscript writing. A.d.C. and S.S. participated in the study design, research supervision, and manuscript writing. J.I.P. participated in the study design. M.K.L.H. participated in research supervision and manuscript revision. C.M. assisted with the experimental procedures and data collection.

*Adv. Sci*. **2019**, *6*, 1801982

DOI: 10.1002/advs.201801982

The above article, published online on May 3, 2019, in Wiley Online Library (https://doi.org/10.1002/advs.201801982), has been retracted by agreement between the authors, the journal Editor‐in‐Chief Kirsten Severing, and Wiley‐VCH GmbH. The retraction has been agreed on following concerns raised by a third party and a subsequent investigation by the corresponding authors. Data depicted in Figure 4 and Figure 5 could not be reproduced in follow‐up experiments. Therefore, the conclusions associated with those figures in the article are considered invalid.

E.S.K. participated in the study design, performed measurements, analyzed the data, compiled the figures and participated in manuscript writing. A.d.C. and S.S. participated in the study design, research supervision, and manuscript writing. J.I.P. participated in the study design. M.K.L.H. participated in research supervision and manuscript revision. C.M. assisted with the experimental procedures and data collection.

